# Syndecan-4 as a Pathogenesis Factor and Therapeutic Target in Cancer

**DOI:** 10.3390/biom11040503

**Published:** 2021-03-26

**Authors:** Jessica Oyie Sousa Onyeisi, Carla Cristina Lopes, Martin Götte

**Affiliations:** 1 Department of Gynecology and Obstetrics, University Hospital Münster, Albert-Schweitzer-Campus 1, D11, 48149 Münster, Germany; 2 Disciplina de Biologia Molecular, Departamento de Bioquímica, Universidade Federal de São Paulo, São Paulo 04039-032, Brazil; cclazevedo@gmail.com; 3 Instituto de Ciências Ambientais, Químicas e Farmacêuticas, Universidade Federal de São Paulo, Diadema 09913-030, Brazil

**Keywords:** syndecan-4, heparan sulfate, cancer, prognosis, biomarker, signal transduction, proteoglycan, metastasis

## Abstract

Cancer is an important cause of morbidity and mortality worldwide. Advances in research on the biology of cancer revealed alterations in several key pathways underlying tumorigenesis and provided molecular targets for developing new and improved existing therapies. Syndecan-4, a transmembrane heparan sulfate proteoglycan, is a central mediator of cell adhesion, migration and proliferation. Although several studies have demonstrated important roles of syndecan-4 in cell behavior and its interactions with growth factors, extracellular matrix (ECM) molecules and cytoskeletal signaling proteins, less is known about its role and expression in multiple cancer. The data summarized in this review demonstrate that high expression of syndecan-4 is an unfavorable biomarker for estrogen receptor-negative breast cancer, glioma, liver cancer, melanoma, osteosarcoma, papillary thyroid carcinoma and testicular, kidney and bladder cancer. In contrast, in neuroblastoma and colorectal cancer, syndecan-4 is downregulated. Interestingly, syndecan-4 expression is modulated by anticancer drugs. It is upregulated upon treatment with zoledronate and this effect reduces invasion of breast cancer cells. In our recent work, we demonstrated that the syndecan-4 level was reduced after trastuzumab treatment. Similarly, syndecan-4 levels are also reduced after panitumumab treatment. Together, the data found suggest that syndecan-4 level is crucial for understanding the changes involving in malignant transformation, and also demonstrate that syndecan-4 emerges as an important target for cancer therapy and diagnosis.

## 1. The Syndecan Family of Cell Surface Heparan Sulfate Proteoglycans

Syndecans are a family of four transmembrane heparan sulfate proteoglycans (syndecan-1, -2, -3 and -4) in mammals. Each syndecan has an extracellular ectodomain that displays low sequence homology, except for the consensus sequences of the attachment sites for carbohydrate chains of the glycosaminoglycan (GAG) type. Syndecans have predominantly heparan sulfate-GAG (HS-GAG) chains attached to the extracellular domain and in the case of Sdc1 and Sdc3, additional chondroitin sulfate GAG chains. HS is a long, unbranched carbohydrate composed of repetitive disaccharide units of N-acetylglucosamine-α-l-iduronic acid/β-d-glucuronic acid, which can be substituted with sulfate residues, thus generating a highly negatively charged biomolecule capable of interacting with numerous ligands relevant to tumor progression [1]. HS chains are synthesized via O-glycosylation in the Golgi apparatus in a series of acetylation and deacetylation and sulfation steps which result in a high degree of structural complexity [2]. The HS chains are covalently linked to serine residues of the core protein via a defined tetrasaccharide linker consisting of glucuronic acid, two galactose residues, and a xylose. The relevance of glycosaminoglycan attachment has been documented in rare human inherited diseases where this GAG attachment site cannot be formed efficiently, resulting in severe developmental defects [3,4]. It has a conserved transmembrane domain, and a short cytoplasmic domain that has two conserved regions, C1 and C2, proximal and distal, respectively, to the membrane, common to all syndecans. The C1 and C2 regions are separated by a variable (V) region unique to each syndecan. Syndecans-1–3 have a restricted tissue distribution, whereas syndecan-4 is expressed ubiquitously [5,6,7]. 

## 2. Syndecan-4

The syndecan-4 has abundant expression in liver, kidney, brain, lung, breast heart, skeletal muscle, skin and small intestine [2] (Gene Expression database (https://www.ncbi.nlm.nih.gov/gene/6385—accessed on 8 January 2021). As outlined in detail below, the broad effects of this molecule are exemplified by its ability to form a connection between the extracellular matrix (ECM) and intracellular signaling cascades and to affect the growth and differentiation of a number of tissues and organs.

### 2.1. Syndecan-4 Membrane Localization, Trafficking and Signaling

Syndecan-4 is important for the interplay between extracellular matrix and cytoplasmatic signaling molecules and scaffolding proteins. It contributes to several outside-in and inside-out signaling events, such as the sequestration and concentration of matrix components, as well as effects on cell–matrix adhesion, endocytosis, exosome biogenesis or cytokinesis [8]. Syndecan-4 is localized to the plasma membrane and also localizes in endocytic compartments such as early endosomes and multivesicular bodies, indicating internalization and trafficking along the endosomal/lysosomal degradation route during muscle cell differentiation. Furthermore, the syndecan-4/syntenin complex is essential for exosome biosynthesis and multivesicular bodies, which give rise to exosomes [9,10]. 

### 2.2. Syndecan-4 as an Extracellular Signaling Interface

**Figure 1:**
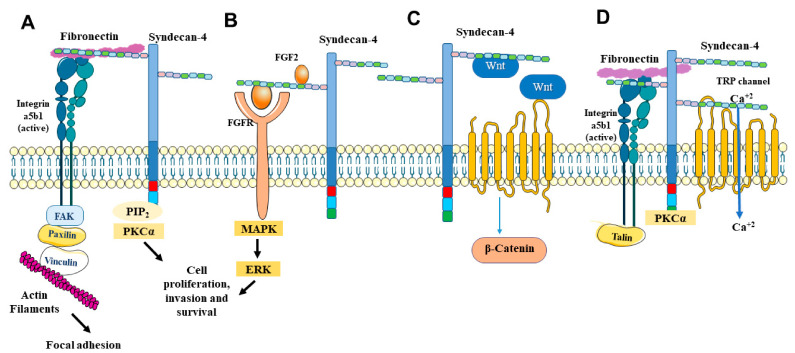
Overview of signaling pathways activated by syndecan-4. (**A**) Syndecan-4 and integrin signaling. (**B**) Syndecan-4 and growth factors. (**C**) Syndecan-4 and Wnt signaling. (**D**) Syndecan-4 and TRPC channels. See text for details.

Through heparan sulfate chains on its extracellular domain, syndecan-4 can bind to various heparin-binding growth factors, chemokines and morphogens [11] (Figure 1). Syndecan-4 is a powerful regulator of FGF-2 signaling and can modulate growth factor responses in multiple cell types. In addition, syndecan-4 is capable of signaling in response to FGF independently of FGF receptor interactions [12,13,14]. Elfenbein and collaborators demonstrated that syndecan-4-mediated modulation of FGF2-induced FGFR1 endocytosis and MAPK signaling represents a previously unappreciated mechanism of crosstalk between the two receptors binding the same ligand [15]. 

Apart from FGFR signaling, syndecan-4 also affects epidermal growth factor (EGF)-mediated signaling, albeit through a different mechanism: It has been shown that EGF-dependent cancer cell migration is mediated through a complex of human epidermal growth factor receptor-1 (EGFR), α6β4 integrin and Sdc4. In this context, syndecan-4 modulates signaling via interactions with the extreme C terminus of the β4 integrin cytoplasmic domain, thereby affecting epithelial cancer cell migration [16]. Moreover, an extracellular site comprising amino acids 87–131 in the ectodomain of syndecan-4 captures EGFR, thus affecting signaling in epithelial cancer cells [17]. Apart from receptor tyrosine kinases, syndecan-4 has also been shown to affect G-protein coupled receptor signaling. For example, syndecan-4 affects hepatoma and HeLa cell motility and invasion by facilitating signaling via chemokines such as RANTES/CCL5 and SDF-1 [18,19], which is in accordance with the role of heparan sulfate in chemokine signaling [20]. The importance of syndecan-4 for the communication between tumor cells and immune cells has also been highlighted in vivo, as syndecan-4-deficient mice show reduced Lewis lung carcinoma growth, less dendritic cell recruitment, and increased recruitment of natural killer cells [21]. Finally, syndecan-4 is also involved in mediating signaling via morphogens such as Wnt. This has been demonstrated in model organisms such as the frog *Xenopus*, where the Wnt modulator R-spondin 3 induces syndecan-4-dependent clathrin-mediated endocytosis of Wnt–receptor complexes, thus affecting morphogenesis [22]. In turn, noncanonical Wnt signaling induces ubiquitination and degradation of syndecan-4 in *Xenopus*, suggesting complex regulatory mechanisms [10]. In a cancer context, silencing of syndecan-4 expression was shown to exhibit an antitumoral effect on human papillary thyroid carcinoma cells by affecting apoptosis and epithelial-to-mesenchymal transition via the Wnt/beta-catenin pathway [23]. Moreover, invasive growth of melanoma cells can be inhibited by syndecan-4 knockdown and rescued by addition of Wnt5a, suggesting an impact of syndecan-4 on this signaling pathway in melanoma [24]. Besides regulating cellular signaling via soluble growth factors and morphogens, syndecan-4 also act as a receptor for ECM molecules. Syndecan-4 facilitates α5β1 integrin binding to its substrate fibronectin, allowing maturation of focal adhesions [25,26]. The engagement of syndecan-4 by fibronectin triggers rapid endocytosis of α_5_β_1_-integrin, due to activation of RhoG [27]. Furthermore, syndecan-4 phosphorylation is a control point for integrin recycling [28]. In addition, through heparan sulphate side chains, syndecan-4 interacts with transglutaminase type 2 (TG2), an extracellular matrix crosslinking enzyme, affecting fibrosis [29].

#### Shedding of the Extracellular Domain

One mechanism by which syndecan-4 regulates its extracellular signaling is the proteolytic cleavage of its intact extracellular domain, in a process called shedding [30]. This cleavage is highly regulated by matrix metalloproteinases (MMPs) and can be accelerated under certain physiological conditions [31].

MMP9 has been shown to cleave syndecan-4 from HeLa cells, human primary macrophages and endothelial cells [32,33]. Our previous work demonstrated that syndecan-4 depletion decreased the expression of MMP3 in endometriosis, resulting in decreased invasive growth [34], and suggesting possible feedback loops. ADAMTS-1, a disintegrin and metalloproteinase with thrombospondin motifs, promotes syndecan-4 shedding, and this shedding disrupts cell adhesion and promotes cell migration [35,36]. 

Syndecan ectodomains can be cleaved by thrombin to produce bioactive fragments. For example, the recombinant ectodomains of human syndecan-3 and syndecan-4 induce significant decreases in endothelial barrier resistance and this involves Rho kinase pathway-mediated F-actin stress fiber formation and VE-cadherin junction disorganization [37]. Modification of the extracellular domain of syndecan-4 with highly flexible glycosaminoglycan side chains makes the receptor ideally suited to the detection of ligands that are dilute or distant from the membrane [38]. 

### 2.3. Intracellullar Signal Transduction Mechanisms

The cytoplasmic domain of syndecan-4 is distinct from the other syndecans in its capacity to bind phosphatidylinositol 4, 5-bisphosphate (PIP_2_) and to activate protein kinase C-alpha (PKC-alpha) [39,40,41]. Syndecan-4 also provokes protein kinase Cα (PKCα) to phosphorylate the transient receptor potential canonical 7 cell membrane channel (TRPC7) that is involved in the regulation of cytosolic calcium levels to control myofibroblast differentiation [42,43]. (Figure 1). Syndecan-4 regulates downstream signaling pathways and the activity of the small GTPase Rac1 which orchestrates actin polymerization in migrating cells [38,44]. In addition, syndecan-4 can regulate the intracellular calcium distribution [43]. Syndecan-4 is known to regulate the organization of cytoskeleton, including focal adhesion and stress fiber formation, and Carvalheiro and coworkers demonstrated that the coupling of vinculin to F-actin demands syndecan-4. The authors showed that syndecan-4 acts as a central mediator that bridges fibronectin, integrin and intracellular components (actin and vinculin) and once silenced, the cytoskeleton protein network is disrupted [45]. Overall, these mechanisms expand the role of syndecan-4 beyond its classical function as a coreceptor for growth factor-mediated receptor tyrosine kinase signaling [12,13,14] and chemokine-mediated signaling via G-protein coupled heptahelical receptors [15,16].

## 3. Syndecan-4 and Cancer

### 3.1. Syndecan-4 Expression in Cancers

Given the multitude of syndecan-4-mediated cellular functions with relevance to tumor progression, it is not surprising that the expression of syndecan-4 is dysregulated in a number of malignant diseases, highlighting its importance as a pathogenesis factor and diagnostic marker (Table 1). In the following section, we will provide an overview of its clinicopathological relevance in a number of tumor entities. 

#### 3.1.1. Breast Cancer

Breast cancer (BC) is a complex heterogeneous form of cancer with numerous genetic alterations and distinct molecular subtypes [55]. According to the American Cancer Society, breast cancer is the most common cancer among women, accounting for nearly one in three cancers diagnosed in women [56]. Several studies have shown that heparan sulfate proteoglycans and specific genes involved in the synthesis and editing of heparan sulfate proteoglycans show altered expression in breast cancer [57,58,59,60,61]. Syndecan-4 is expressed in normal human mammary epithelium, and was initially described as being overexpressed in an estrogen receptor-negative, highly proliferative breast carcinoma subtype [46]. However, in a study on duplicate samples of benign and malignant breast cancer cases, syndecan-4 expression was found to be correlated with positive estrogen and progesterone receptor status, and found to exhibit an expression pattern distinct from syndecan-1, suggesting divergent pathobiological roles for these proteoglycans [62]. Besides being the second most abundant HSPG produced by most breast carcinoma cell lines [62], syndecan-4 is involved in membrane fixation of LL-37 and its pro-migratory effect in breast cancer cells [63]. Moreover, in vivo, targeting of syndecan-4 in murine 4T1 breast cancer cells inhibited the formation of early bone metastases [64]. Interestingly, a study demonstrated that estrogen receptor beta (ERβ) silencing in MDA-MB-231 breast cancer cells induces the expression of syndecan-4 [65], suggesting endocrine regulation of syndecan-4 in this tumor entity. This view is supported by investigations of menstrual cycle-dependent expression changes in healthy breast tissue, where syndecan-4 mRNA expression was significantly lower among parous women in the progesterone-dominated luteal phase compared to the estrogen-dominated follicular phase [66]. Apart from steroidal mechanisms, inhibition of the receptor tyrosine kinase IGFR was shown to downregulate syndecan-4 levels in estrogen receptor-positive breast cancer cells via an endocytic mechanism [67].

#### 3.1.2. Colon Cancer

Heparan sulfate proteoglycans, as well as heparan sulfate remodeling enzymes, are molecules involved in colorectal cancer tumorigenesis [68]. In normal epithelial cells and tissues, the expression level of syndecan-4 is high, however, syndecan-4 is significantly reduced in highly metastatic colon carcinoma cells (KM1214) [47,69]. Hypoxia is one of the factors regulating syndecan-4 expression in human colon cancer cells, as it can induce its expression, along with alpha 5 integrin [70]. Interestingly, hetero-oligomerization with syndecan-2 reduces both syndecan-4-dependent PKCα activation and cell adhesion and syndecan-2-mediated migration and anchorage-independent growth in colon cancer cells, suggesting a functional interplay of syndecans in tumor progression [71]. Moreover, the recombinant syndecan-4 ectodomain is capable of inducing the expression of the epidermal growth factors erb-b2 and erb-b3 in colon cancer cells, suggesting a regulatory crosstalk between these receptor tyrosine kinases and the proteoglycan [68]. In turn, experimental lung metastasis of the murine colon cancer cell line MC-38 resulted in an induction of syndecan-4 expression in blood vessels at the metastatic site, suggesting a possible role for this proteoglycan in the metastatic niche [72].

#### 3.1.3. Glioma 

Human glioma is the most common type of primary brain tumor worldwide. Using proteomics analysis, a recent study showed the pull-down of multiple cancer-related proteoglycans with key roles in the pathogenesis of glioma [73]. All malignant glioma cell lines and glioblastoma specimens expressed all types of syndecans at the mRNA level. Syndecan-4 is highly expressed on the surface of glioma cells [48]. Interestingly, syndecan-4 mRNA expression has been indicated as a novel marker for the prediction of glioblastoma multiforme patients’ response to treatment with the WT1 peptide vaccine [74], and its expression is altered in pediatric astrocytoma [75]. 

#### 3.1.4. Liver Cancer

Liver cancer is the most frequent cause of cancer deaths across the globe [76]. Alterations in proteoglycan expression interfere with the physiologic function of the liver on several levels and, in addition, this affects cancer cell signaling pathways, facilitating tumorigenesis [77,78]. Syndecan-4 is expressed in human normal liver [79]. In both hepatocellular carcinoma (HCC) and cholangiocarcinoma, increased levels of syndecan-4 were found [49]. Notably, studies in a susceptible mouse model of Moloney murine leukemia, virus infection demonstrated that provirus integration at a site upstream of the first exon of the syndecan-4 gene resulted in particularly fast-growing hepatocellular carcinomas [80]. Moreover, syndecan-4 plays an important coreceptor role in the effects of the chemokine SDF-1 on human hepatoma cell growth, migration, and invasion [81].

#### 3.1.5. Melanoma

Melanoma is a highly aggressive skin cancer [82]. Along with beta 3 integrin and WNT5A, syndecan-4 is part of a gene signature characteristic for metastatic disease in melanoma [83]. Indeed, syndecan-4 is an important component of the Wnt5A autocrine signaling loop and its overexpression is correlated to increased metastatic potential in melanoma patients. In addition, the knockdown of syndecan-4 caused decreases in cell invasion of metastatic melanoma cells [24]. Moreover, a study has shown that inhibition of syndecan-binding protein syntenin-1 (SDCBP) expression by siRNA impaired the ability of uveal melanoma cells to migrate in a wound-healing assay [84]. 

In contrast, a different study demonstrated that reduction in syndecan-4 expression in melanoma cells resulted in downregulation of FGF-2 signaling, leading to an increase in tumor cell motility and decreased adhesion to fibronectin, demonstrating a regulatory role of syndecan-4 on these cell functions [85]. Of note, syndecan-4 is required for the activating function of latent heparanase in the activation of VLA4 integrin in melanoma cells [86]. Finally, syndecan-4 overexpression significantly reduces the migration of A375 melanoma cells, whereas its siRNA knockdown enhanced their migration, consistent with the observation that syndecan-4 overexpression reduced lung and popliteal lymph node metastasis of B16F10 melanoma cells in mice. Notably, syntenin overexpression could compensate for the effect of syndecan-4 depletion, suggesting functional interactions [87]. 

#### 3.1.6. Neuroblastoma

Neuroblastoma is a pediatric malignancy that originates from the neural crest. Previous works have shown that extracellular matrix components contribute to tumor progression in neuroblastoma [88,89]. Using microarray dataset analysis, Knelson and collaborators demonstrated that syndecan-4 expression is reduced in neuroblastoma in comparison with benign neuroblastic tumors and is high in the Schwannian stroma [50].

#### 3.1.7. Osteosarcoma

Osteosarcoma is the most common malignant bone tumor in young adults and children [90,91]. Pathogenesis of osteosarcoma implicates qualitative and quantitative changes in the proteoglycans [92,93]. A study has shown that syndecan-4 expression is upregulated in high-grade osteosarcoma when compared to other tissues. In addition, its overexpression was significantly associated with a larger tumor size, distant metastasis and poor overall survival [51]. The expression of syndecan-4 on osteosarcoma cell lines in vitro can be induced by the cytokines lL-1b and IL-6, but not by the osteotropic hormones parathyroid hormone (PTH(1–34)), and 1,25(OH)2-vitamin D3 [94]. Mechanistically, syndecan-4 mediates tumorigenic properties of osteosarcoma cells via cell surface interactions with autotaxin-β [64].

#### 3.1.8. Testicular Germ Cell Tumors

Testicular germ cell tumors are the most common malignancy of young adult males [95,96]. They are classified into seminomatous germ cell tumors (testicular germ cell tumors, TGCTs) and nonseminomatous germ cell tumors (NSGCTs), the latter being either undifferentiated (embryonal carcinoma) or differentiated (teratoma, yolk sac tumor and choriocarcinoma) [97]. In both seminomatous testicular germ cell tumors (TGCTs) and nonseminomatous germ cell tumors (NSGCTs), significantly increased expression of syndecan-4 was detected in tumor cells. Syndecan-4 is differentially expressed in seminomas and NSGCTs and might be a useful marker [52]. Studies on rat Sertoli cell development have demonstrated that syndecan-4 expression in healthy testes is regulated by protein kinase C, follicle-stimulating hormone and the second messenger cAMP, providing possible avenues for pharmacological intervention in the context of malignant disease [98,99].

#### 3.1.9. Papillary Thyroid Cancer

Papillary thyroid cancer is the most common type of thyroid cancer [100,101]. To obtain proteomic profiles from various thyroid cancer cell lines that represent the range of thyroid cancers of follicular cell origin, a study used a proteomics strategy targeting cell surface and secreted proteins and identified syndecan-1 and syndecan-4 as glycoproteins uniquely expressed by the various thyroid cancer cell lines [102]. Using a microarray, two recent studies have shown that syndecan-4 expression levels among the papillary thyroid cancer tissues are higher than that in normal thyroid tissues [103,104]. Interestingly, syndecan-4 gene silencing represses EMT, and enhances cell apoptosis by suppressing the activation of the Wnt/β-catenin signaling pathway in human papillary thyroid carcinoma [23].

#### 3.1.10. Kidney Cancer

Renal cell carcinoma, also known as hypernephroma, renal adenocarcinoma or Grawitz tumor, is the most common malignant type of kidney cancer [105]. Renal cell carcinoma is characterized by profound changes in cellular metabolism such as glucose and glutamine utilization, lipid metabolism and mitochondrial function [106]. A study demonstrated that metastatic Caki-1 and ACHN cells (human renal adenocarcinoma) expressed higher levels of syndecan-4 mRNA than primary renal cell carcinoma cell lines. The authors concluded that upregulation of syndecan-4 mRNA plays an important role in the development of renal cell carcinoma and advanced forms of the disease with metastasis [53]. In contrast, a study utilizing data from the Human Protein Atlas dataset assigned a positive prognostic value to high syndecan-4 protein expression in renal cell carcinoma [107]. Different methodological approaches, such as the assessment of syndecan-4 mRNA vs. protein levels, may account for the discordant results of these studies.

#### 3.1.11. Bladder Cancer

Bladder cancer is the most common malignancy of the urinary tract and is common in women and the fourth most common malignancy in men [108,109]. The expression of syndecan-1, -2 and -3 is decreased while syndecan-4 is increased in bladder cancers compared to normal tissues [54,110]. Functionally, interactions of syndecan-4 and angiomodulin have been found to be responsible for the formation of cord-like structures in the human bladder carcinoma cell line ECV-304 [111]. 

### 3.2. Syndecan-4 in Cancer Biology

The dysregulated expression of syndecan-4 in numerous tumor entities (see Table 1) suggests a possible mechanistic contribution to cancer progression. Indeed, proteoglycans are capable of modulating virtually all hallmarks of cancer [112,113,114]. In the following section, we will highlight the role of syndecan-4 in selected tumor-associated cellular functions.

#### 3.2.1. Survival

Disruption of cell–matrix attachment results in a loss of prosurvival signals and culminates in programmed cell death, referred to as anoikis [115]. Tumor cells often resist anoikis, survive and grow in the absence of anchorage to the extracellular matrix (ECM). In our previous work, we have demonstrated that the acquisition of anoikis resistance by blocking adhesion to the substrate upregulates syndecan-4 expression in endothelial cells and syndecan-4 gene silencing reverses the transformed phenotype of anoikis-resistant endothelial cells [116,117]. The Ras/Raf/MAPK (MEK)/ERK pathway plays a crucial role in the survival and development of tumor cells [118,119]. Neel and collaborators demonstrated that SDC4-ROS1 and SLC34A2-ROS1 fusion oncoproteins reside on endosomes and activate the MAPK pathway. Moreover, they showed that knockdown of these fusion proteins resulted in suppression of the RAS/MAPK pathway [120]. In addition, knockdown of syndecan-4 in human papillary thyroid carcinoma cells promoted apoptosis via the Wnt/beta catenin pathway [23]. Moreover, knockdown of syndecan-4 reduces macrophage cell surface TG2 activity and apoptotic cell clearance [121]. Finally, association of the chemokine SDF-1 with syndecan-4 increases the resistance of hepatoma cells to TNF-alpha-induced apoptosis [80].

#### 3.2.2. Proliferation

Proliferation is an important part of cancer development and progression [122]. The Ras–Raf–MEK–ERK signaling cascade is crucial for controlling this process [123]. Several studies have shown that syndecan-4 signaling can lead to ERK activation and induce cell proliferation [124,125]. Syndecan-4 promotes cytokinesis in a phosphorylation-dependent manner in MCF-7 breast adenocarcinoma cells, which shed the ectodomain of syndecan-4 periodically in a cell cycle-dependent way, reaching the maximum at the G2/M phase [126]. Several works have demonstrated that syndecan-4 gene silencing suppresses the cell cycle progression, decreasing the transition from G1 to S phase and decreasing the levels of cyclin D1 and cyclin E in different cancer cell lines [117,127,128]. Recently, it was demonstrated that the prometastatic integrin-interacting factor autotaxin-beta promotes osteosarcoma cell proliferation via a mechanism that requires a physical interaction with syndecan-4 [64], expanding the range of mechanisms by which syndecan-4 regulates tumor cell growth.

#### 3.2.3. Adhesion

Tumor cells often show a decrease in cell–cell and/or cell–matrix adhesion [129]. Besides that, changes in cell adhesion molecules play a causal role in tumor dissemination [130]. Syndecan-4 is an important regulator of cell adhesion [11]. α_V_β_1_ integrin and syndecan-4 are key players of the interaction with vitronectin in bladder cancer cells. Although these surface receptors share a similar role, the energy landscapes of single molecular complexes reveal higher (integrins) and lower (syndecans) energy barriers. The shape of the energy landscape agrees with the binding site structures of both complexes [131]. Notably, epithelial cell spreading depends on the interaction of syndecan-4 with integrins, as β4 integrin mutants deficient in syndecan-4 recognition act in a dominant negative manner to block EGFR-dependent cell spreading [16]. Recent works identified PAR-3 as a syndecan-4-binding protein and the syndecan-4/PAR-3 signaling complex participates in Thy-1/CD90-induced focal adhesion disassembly in mesenchymal cells [132].

#### 3.2.4. Cell Migration 

There are several mechanisms by which syndecan-4 contributes to tumor cell migration. Syndecan-4 promotes cell migration in a variety of cells. Syndecan-4 is involved in membrane fixation of cathelicidin LL-37 and its promigratory effect in breast cancer cells [63]. It is also involved in RANTES/CCL5-induced migration and invasion of human hepatoma cells [19]. Ochieng and collaborators have demonstrated that knockdown of syndecan-4 significantly attenuated the invasive capacity and uptake of labeled exosomes and FNH (fetuin-A and histones) nanoparticles of LN229, a highly aggressive glioblastoma cell line [133]. Similar results were found in anoikis-resistant endothelial cells, and syndecan-4 silencing led to downregulation of the invasive capacity of anoikis-resistant endothelial cells [117]. Syndecan-4 is also capable of modulating the effect of other matrix constituents in tumor cell migration: In breast cancer, the protease ADAMTS-15 reduces cell migration on fibronectin and laminin matrices. Notably, the inhibitory effect could be rescued by knockdown of syndecan-4, suggesting a mechanistic role in this context which is worthy of further exploration [134].

#### 3.2.5. Tumor Angiogenesis

Angiogenesis is an important step for cancer growth and progression [135]. The ability of syndecan-4 to regulate angiogenesis, the formation of new blood vessels from an existing vasculature, is suggested by the observation that the codelivery of proteoliposomes with FGF-2 increased the cellular uptake, trafficking and nuclear localization of the growth factor. These alterations in cellular signaling, trafficking and nuclear localization led to increased proliferation, migration and angiogenic differentiation in response to FGF-2 treatment [13]. Moreover, the ability of endothelial tube formation in matrigel is reduced upon syndecan-4 silencing, which was partially attributed to the role of syndecan-4 in coupling viculin to F-actin, and to connecting actin filopodial protrusions to vascular endothelial cadherin-rich junctions [45].

In endometriosis, a disease characterized by invasive growth of endometrial tissue at ectopic sites, syndecan-4 is upregulated along with constituents of TGF-beta signaling, and modulates invasion via regulation of MMP and RAC1 expression [34,136]. Moreover, in a mouse model of pathological lymphangiogenesis, syndecan-4 was the predominant heparan sulfate proteoglycan in mouse lymphatic endothelia, and showed a VEGF-C-induced association with VEGF receptor-3 at the lymphatic cell surface. Notably, syndecan-4-deficient mice showed an impaired pathological lymphangiogenesis in this model, suggesting a possible coreceptor function for VEGF-C [21]. Recent findings document not only a role for syndecan-4 in lymphangiogenesis, but also in classical VEGFA-mediated angiogenesis: Syndecan-4-deficient mice show reduced angiogenesis not only in a model of diabetic retinopathy, but also in a melanoma model of tumor angiogenesis. The impact of syndecan-4 on these processes was shown to involve a mechanism by which syndecan-4 localized at endothelial cell junctions interacts with vascular endothelial cadherin, and participates in its internalization in response to VEGFA [137].

### 3.3. Syndecan-4 as a Target for Anticancer Drugs

The dysregulation of syndecan-4 in various cancers, and its mechanistic contribution to multiple steps of tumor progression, mark it as an attractive potential target for cancer therapy. Several studies have demonstrated that therapeutics currently used in clinics have an impact on syndecan-4 expression and its functions (Table 2).

#### 3.3.1. Trastuzumab

Trastuzumab (Herceptin^®^) is a humanized recombinant monoclonal antibody (mAb) of the immunoglobulin G1 type, approved by the FDA for treatment of breast and gastric cancer with overexpression of ErbB2 (HER2) [141,142]. Recently, we demonstrated that trastuzumab reduces syndecan-4 expression in anoikis-resistant endothelial cells and this interaction controls cellular events, such as proliferation, adhesion and angiogenesis, in these cells [138].

#### 3.3.2. Panitumumab

Panitumumab is a human monoclonal antibody (pMAb) approved by the FDA in 2007, which inhibits epidermal growth factor receptor (EGFR). Panitumumab is used for the treatment of patients with metastatic colorectal cancer. A first study reported that panitumumab (pmAb) significantly decreases the expression of syndecan-4 [139]. 

#### 3.3.3. Bisphosphonate Zoledronic Acid (ASCO) 

Bisphosphonate zoledronic acid (ASCO) inhibits osteoclast-mediated bone resorption. It has been approved for treatment of patients with advanced lung cancer, renal cancer and other solid tumors with bone metastases or multiple myeloma and for the management of tumor-induced hypercalcemia [143,144]. Interestingly, Dedes and collaborators demonstrated that syndecan-4 expression is upregulated upon treatment with zoledronate [140].

Overall, these data suggest that syndecan-4 expression and function is already modulated by existing anticancer drugs, and therefore part of the therapeutic response. Syndecan-4 has been targeted or utilized as part of therapeutic approaches in nonmalignant diseases, and this knowledge could be utilized in a cancer context in the future: For example, antibody-mediated inhibition of syndecan-4 has been proposed as a treatment for osteoarthritis [145,146]. Moreover, syndecan-4 enhances uptake of liposomes for therapeutic gene delivery [147]. Notably, binding of the cell-penetrating peptide Xentry to syndecan-4 has been utilized to target therapeutics to melanoma cells in vitro [148]. While not specific to syndecan-4 alone, a targeting of the heparan sulfate moiety may expand the range of approaches hampering syndecan-4 function in cancer [149,150].

## 4. Conclusions

In this review, we demonstrated that changes in the expression of syndecan-4 contribute to the development and progression of cancer, and have a diagnostic and prognostic value in numerous tumor entities. Besides that, we showed that syndecan-4 has an important role in the hallmarks of cancer, modulating multiple steps of tumor progression, including unlimited cell proliferation, resistance to apoptosis, invasive growth and metastasis, tumor angiogenesis and tumor-associated inflammation. Syndecan-4 mediates these processes as a signaling interface at the cell surface, acting as a classical heparan sulfate coreceptor for soluble ligands such as growth factors and chemokines, but also via interactions of its protein moiety with growth factor receptors and integrins. The data reviewed in this article support that a targeting of syndecan-4, or a modulation of its expression using already available drugs, may be promising strategy for the treatment of different types of cancers.

## Figures and Tables

**Table 1:** Syndecan-4 expression in different types of cancer.

Cancer Type	Syndecan-4 Expression	References
Breast Cancer (Estrogen receptor-negative)	Overexpressed	[46]
Colorectal	Reduced	[47]
Glioma	Overexpressed	[48]
Liver	Overexpressed	[49]
Melanoma	Overexpressed	[24]
Neuroblastoma	Reduced	[50]
Osteosarcoma	Overexpressed	[51]
Testicular	Overexpressed	[52]
Papillary Thyroid Carcinoma	Overexpressed	[23]
Kidney	Overexpressed	[53]
ladder	Overexpressed	[54]

**Table 2:** Effects of anticancer drugs on syndecan-4 expression in different cell models.

Anticancer Drug	Cell Type	Biological Effects	References
Trastuzumab (Herceptin^®^)	Anoikis-resistant endothelial cells	Decreases syndecan-4 expression	[138]
Panitumumab (Vectibix^®^)	Colon cancer		[139]
Bisphosphonate Zoledronic acid (ASCO)	Breast cancer	Syndecan-4 upregulation	[140]

## Data Availability

Not applicable.
